# Influence of serum IL-36 subfamily cytokines on clinical manifestations of asthma

**DOI:** 10.1016/j.jacig.2025.100419

**Published:** 2025-01-18

**Authors:** Yuki Hoshino, Tomoyuki Soma, Kazuyuki Nakagome, Reina Ishii, Tatsuhiko Uno, Kazuki Katayama, Hidetoshi Iemura, Erika Naitou, Takahiro Uchida, Yoshitaka Uchida, Hidetoshi Nakamura, Makoto Nagata

**Affiliations:** Department of Respiratory Medicine and Allergy Center, Saitama Medical University, Iruma-gun, Saitama, Japan

**Keywords:** Asthma, IL-36, type 2, non–type 2, IL-6, pulmonary function, acute exacerbation

## Abstract

**Background:**

The IL-36 subfamily, a member of the IL-1 superfamily, is thought to promote type 2 (T2) and non-T2 inflammation and involved in autoimmune and airway disease progression. However, its role in asthma remains unclear.

**Objective:**

We sought to determine the contribution of the IL-36 subfamily to the clinical manifestation of asthma.

**Methods:**

The levels of serum IL-36α, IL-36β, and IL-36γ, recognized as IL-36 subfamily agonists, and IL-36 receptor antagonist (IL-36Ra) and IL-38, recognized as IL-36 subfamily antagonists, were measured by ELISA in 110 asthma patients (55 with nonsevere and 55 with severe asthma) aged ≥20 years and 31 healthy individuals. The association of IL-36 with clinical indices and inflammatory mediators was examined. The characteristics of high and low IL-36 subgroups were explored.

**Results:**

IL-36α, IL-36γ, and IL-36Ra levels were significantly higher in asthma patients, especially patients with severe asthma, than in healthy controls. The high IL-36γ group exhibited lower Asthma Control Test scores (*P* = .01), more frequent asthma exacerbations (AEs), and higher hazard ratio for AEs. The high IL-36Ra group exhibited higher values of forced expiratory volume in 1 second, more frequent severe AEs, and higher hazard ratio for severe exacerbations. The IL-36 cytokine levels, except for IL 36α, were positively correlated with IL-6, IL-13, IL-17, and/or IFN-γ levels. IL-36Ra was positively correlated with age-adjusted forced expiratory volume and forced vital capacity.

**Conclusion:**

A systemically high IL-36 level is associated with asthma severity and with both T2 and non-T2 cytokines, and it implies poor condition and enhancement of risk of AEs in asthma patients.

Asthma is an allergic inflammatory disease driven by type 2 (T2) and non-T2 immune pathways, resulting in diverse phenotypes and varying levels of severity.^1^, ^2^, ^3^ IL-33, thymic stromal lymphopoietin, and IL-25, primarily derived from bronchial epithelial cells, regulate IL-4, IL-5, and IL-13, contributing to the development of treatment-resistant T2 inflammatory asthma.^4^^,^^5^ The non-T2 immune pathway drives composite inflammation, partially interacting with the T2 pathway.^6^, ^7^, ^8^, ^9^, ^10^

The IL-36 subfamily, part of the IL-1 superfamily, includes 3 agonists (IL-36α, IL-36β, and IL-36γ) and 2 antagonists (IL-36 receptor antagonist [IL-36Ra] and IL-38).^11^, ^12^, ^13^ IL-36 cytokines are predominantly expressed by epithelial cells, keratinocytes, fibroblasts, macrophages, T cells, and neutrophils and are released in response to external stimuli.^12^^,^^13^ They share a common receptor, IL-36R, which is expressed in multiple organs and immune cells.^13^

IL-36α, IL-36β, and IL-36γ promote T_H_1 cytokine and chemokine production, leading to neutrophilic inflammation in mice.^11^, ^12^, ^13^, ^14^ In addition, they are involved in the innate immune response and contribute to tissue injury.^10^^,^^15^^,^^16^ Conversely, IL-36Ra and IL-38 inhibit T2 and non-T2 inflammatory cascades.^11^, ^12^, ^13^^,^^17^, ^18^, ^19^ IL-36 cytokines are implicated in the progression of autoimmune and inflammatory diseases, including psoriasis, rheumatoid arthritis, and inflammatory bowel disease.^20^, ^21^, ^22^

Several small-scale studies have denoted the involvement of IL-36 cytokines in allergic rhinitis, chronic rhinosinusitis, and chronic obstructive pulmonary disease (COPD), in which increased *IL-36α*, *IL-36β*, and/or *IL-36γ* mRNA expression and protein levels were associated with disease severity.^23^, ^24^, ^25^, ^26^, ^27^, ^28^, ^29^ Additionally, *IL-36* mRNA expression in nasal tissues has been correlated with eosinophilic and neutrophilic inflammation in chronic rhinosinusitis.^28^ Previous studies showed IL-36 cytokines involvement in allergic airway diseases, associating IL-36 cytokines with neutrophils and several inflammatory cytokines in the sputum of patients with mild asthma^30^ and elevated serum IL-36 levels in allergic rhinitis with asthma.^29^ However, its systemic contribution to pathophysiology and clinical features of asthma dose is not fully elucidated. We aimed to determine the influence of serum IL-36 levels on asthma’s clinical manifestations, including disease control.

## Methods

### Patients and study design

This single-center cross-sectional observational study involved Japanese patients with asthma and healthy volunteers aged >20 years, recruited from the Allergy Center of Saitama Medical University Hospital between March 2020 and December 2023. Asthma diagnosis followed the Japanese Society of Allergology guidelines,^31^ characterized by typical asthmatic symptoms or exacerbation episodes, and bronchial reversibility or airway hyperresponsiveness (see the Methods section in this article’s Online Repository available at www.jaci-global.org). Severe asthma was classified according to the American Thoracic/European Respiratory Society (ATS/ERS) guidelines.^32^ Individuals with severe asthma exacerbation (SAE) or respiratory infection within 4 weeks before study enrollment; those with other lung diseases, malignancy, renal failure, or cardiac failure; and pregnant women were excluded from the study. All participants underwent blood tests, pulmonary function tests, and measurement of fractional exhaled nitric oxide (Feno), and they completed the Asthma Control Test (ACT). Written informed consent was obtained from all participants. The study received ethical approval from the institutional review board of Saitama Medical University Hospital (approval 19132.02) and was registered in the University Hospital Medical Information Network Clinical Trials Registry (UMIN 000049253).

### Measurement of serum IL-36 cytokines and other cytokines

Serum IL-36α, IL-36β, IL-36γ, IL-36Ra, and IL-38 levels were measured using ELISA kits (R&D Systems, Minneapolis, Minn). The lower detection limits were 12.5, 12.5, 18.8, 93.8, and 31.2 pg/mL for IL-36α, IL-36β, IL-36γ, IL-36Ra, and IL-38, respectively. Serum IL-4, IL-5, IL-6, IL-8, IL-13, IL-17, IFN-γ, and TNF concentrations were measured using ELISA kits (R&D Systems) or Bio-Plex human 17 cytokine assay kits and its suspension array system (Bio-Rad, Mississauga, Canada). These kits were used as per manufacturer’s instructions. The lower detection limits of the kits, instead of any undetected values, were adopted for analysis in this study.

### Pulmonary function and Feno

Eligible participants underwent pulmonary function and Feno testing according to ATS recommendation.^33^

### Patient stratification by serum IL-36 cytokine level

We determined the reference values of serum IL-36 cytokines to classify asthma patients with and without elevated IL-36 levels on the basis of the distribution of these values in healthy volunteers. The patients were stratified into 2 subgroups using these reference values: asthma patients with serum IL-36 levels below (low serum subgroup) and above (high serum subgroup) the lower limit of detection. AE and SAE were defined according to the ATS/ERS guidelines.^34^ More details are provided in the Methods section in the Online Repository.

### Data analysis

JMP v13.2.0 (SAS Institute, Cary, NC) and SPSS v26.0 (IBM, Armonk, NY) software were used for data analysis. Values with normal and nonnormal distributions were presented as means (standard deviations) and medians (interquartile ranges), respectively. The serum IL-36 levels were log transformed for statistical analysis. Continuous variables with a normal distribution were analyzed by Student *t* test and analysis of variance, and those with a nonnormal distribution by Mann-Whitney *U* test and Kruskal-Wallis test. Categorical variables were analyzed by chi-square test. Correlations between several parameters were assessed by Spearman correlation test, and, if appropriate, partial correlation analysis was adjusted for age as a covariate. The time to the first AE during 6 months after baseline was examined by log-rank test. Hazard ratios (HRs) for the first AE were examined by Cox proportional hazards model adjusted for age, sex, and potential explanatory variables with *P* < .1 in the univariate analysis, which were identified in the univariable Cox proportional hazards model.

## Results

### Patient characteristics

Forty-one healthy controls participated in the study, of whom 31 were finally enrolled after excluding 10 participants: 3 patients were excluded because they had a history of childhood asthma, and 7 patients had high Feno levels. A total of 130 asthma patients were enrolled, but 20 were excluded, as follows: autoimmune diseases in 3, consent withdrawal in 7, and COPD diagnosis in 10. Finally, 110 patients were included in the analysis. Asthma patients had higher chronic sinusitis rates, total IgE levels, blood eosinophil counts, and Feno levels, and lower pulmonary functions than healthy controls (Table I). Severe asthma patients had higher inhaled corticosteroid doses and rates of concomitant controller use and lower total IgE levels and lower pulmonary function, except for forced expiratory volume in 1 second (FEV_1_)/forced vital capacity (FVC), than those without severe asthma (Table I). There were no differences in asthma duration, prevalence of upper airway comorbidities, oral corticosteroid receipt rates, daily oral corticosteroid doses, ACT scores, blood biomarker levels, and Feno levels between asthma patients and healthy controls and between patients with and without severe asthma.

**Table I:** Demographic and clinical characteristics of study participants

Characteristic	Healthy control subjects	Patients with asthma			*P* value	
		Total	Nonsevere	Severe	Healthy vs all patients	Nonsevere vs severe asthma
No. of subjects	31	110	55	55		
Age (years)	53.0 (49.0-65.0)	61.5 (48.7-71.2)	62.0 (42.0-71.0)	61.0 (52.0-72.0)	NS	NS
Sex, no. (%)						
Female	12 (38.7)	62 (56.3)	28 (50.9)	34 (61.8)	NS	NS
Male	19 (61.3)	48 (43.7)	27 (49.1)	21 (38.2)		
Smoking history, no. (%)						
None	18 (58.1)	55 (50.0)	30 (54.6)	25 (45.4)	NS	NS
Past	12 (38.7)	53 (48.2)	23 (41.8)	30 (54.6)		
Present	1 (3.2)	2 (1.8)	2 (3.6)	0		
Body mass index (kg/m^2^)	22.9 (2.5)	23.3 (3.5)	23.3 (3.3)	23.4 (3.8)	NS	NS
Duration of asthma (years)	NA	10.0 (2.4-17.5)	10.0 (2.7-16.0)	9.0 (2.0-21.0)	NA	NS
Allergic rhinitis, no. (%)	14 (45.2)	66 (60.0)	33 (60.0)	33 (60.0)	NS	NS
Chronic sinusitis, no. (%)	2 (6.4)	30 (27.2)	12 (21.8)	18 (32.7)	.01∗	NS
Medication						
ICS dose (μg/d)	NA	500 (400-800)	400 (200-400)	800 (400-800)	NA	<.0001∗
LABA, no. (%)	NA	95 (86.3)	41 (74.5)	54 (98.1)	NA	.0003∗
LTRA, no. (%)	NA	63 (57.2)	22 (40.0)	41 (74.5)	NA	.0003∗
LAMA, no. (%)	NA	32 (29.0)	7 (12.7)	25 (45.4)	NA	.0002∗
Theophylline, no. (%)	NA	25 (22.2)	5 (9.1)	20 (36.4)	NA	.0006∗
Oral corticosteroids, no. (%)	NA	10 (9.1)	3 (5.4)	7 (12.7)	NA	NS
Oral corticosteroids (mg/d), median (min-max)	NA	0 (0-10)	0 (0-5)	0 (0-10)	NA	NS
Biologics, no. (%)†	NA	12 (10.9)	3 (5.4)	9 (16.3)	NA	NS
ACT (score)	NA	23.5 (20.0-25.0)	24.0 (21.0-25.0)	23.0 (17.0-25.0)	NA	NS
Patient with AEs, no. (%)‡	NA	12 (10.9)	3.0 (5.4)	9.0 (16.3)	NA	NS
No. of AEs, median (min-max)‡	NA	0.0 (0.0-5.0)	0.0 (0.0-1.0)	0.0 (0.0-5.0)	NA	NS
Patient with SAEs, no. (%)‡	NA	6 (5.4)	1.0 (1.8)	5.0 (9.1)	NA	NS
No. of SAEs, median (min-max)‡	NA	0.0 (0.0-5.0)	0.0 (0.0-1.0)	0.0 (0.0-5.0)	NA	NS
Total IgE (IU/L)	47.2 (31.2-120.0)	231.5 (71.7-755.2)	293.0 (69.4-883.0)	181.0 (72.0-518.0)	<.0001∗	NS
Blood eosinophil count (/μL)	99 (49-169)	217 (126-473)	190 (148-356)	280 (102-581)	.0002∗	NS
Feno (ppb)	15.0 (11.7-21.2)	26.0 (16.5-47.5)	25 (15-33)	30 (17-93)	.0001∗	NS
Pulmonary function						
FEV_1_ (L)	2.85 (0.64)	2.08 (0.75)	2.29 (0.7)	1.86 (0.75)	<.0001∗	.001∗
FEV_1_ (%)	94.6 (12.3)	83.4 (18.8)	88.1 (17.6)	78.6 (19.0)	<.0001∗	.01∗
FVC (L)	3.51 (0.81)	2.77 (0.87)	3.02 (0.88)	2.51 (0.78)	<.0001∗	.001∗
FVC (%)	95.4 (9.99)	90.4 (15.4)	94.1 (15.5)	86.7 (14.6)	NS	.01∗
FEV_1_/FVC (%)	82.2 (5.67)	74.4 (10.4)	76.1 (9.15)	72.8 (11.4)	<.0001∗	NS

Parametric data are expressed as medians (standard deviations) and nonparametric data as medians (25-75%) unless otherwise indicated. ICS dose values are described as converted to corresponding dose to fluticasone propionate: 2 mg beclomethasone = 2 mg budesonide = 1 mg mometasone = 0.2 mg fluticasone furoate = 1 mg fluticasone propionate.

*ICS,* Inhaled corticosteroid; *LABA,* long-acting β-agonist; *LAMA,* long-acting muscarinic antagonist; *LTRA,* leukotriene receptor antagonist; *NA,* not applicable; *NS,* not significant.

∗ Statistically significant.

† Biologics include 1 omalizumab, 5 mepolizumab, 5 benralizumab, and 1 dupilumab.

‡ AE occurred for 6 months.

### Serum IL-36 levels of asthma patients

**Fig 1:**
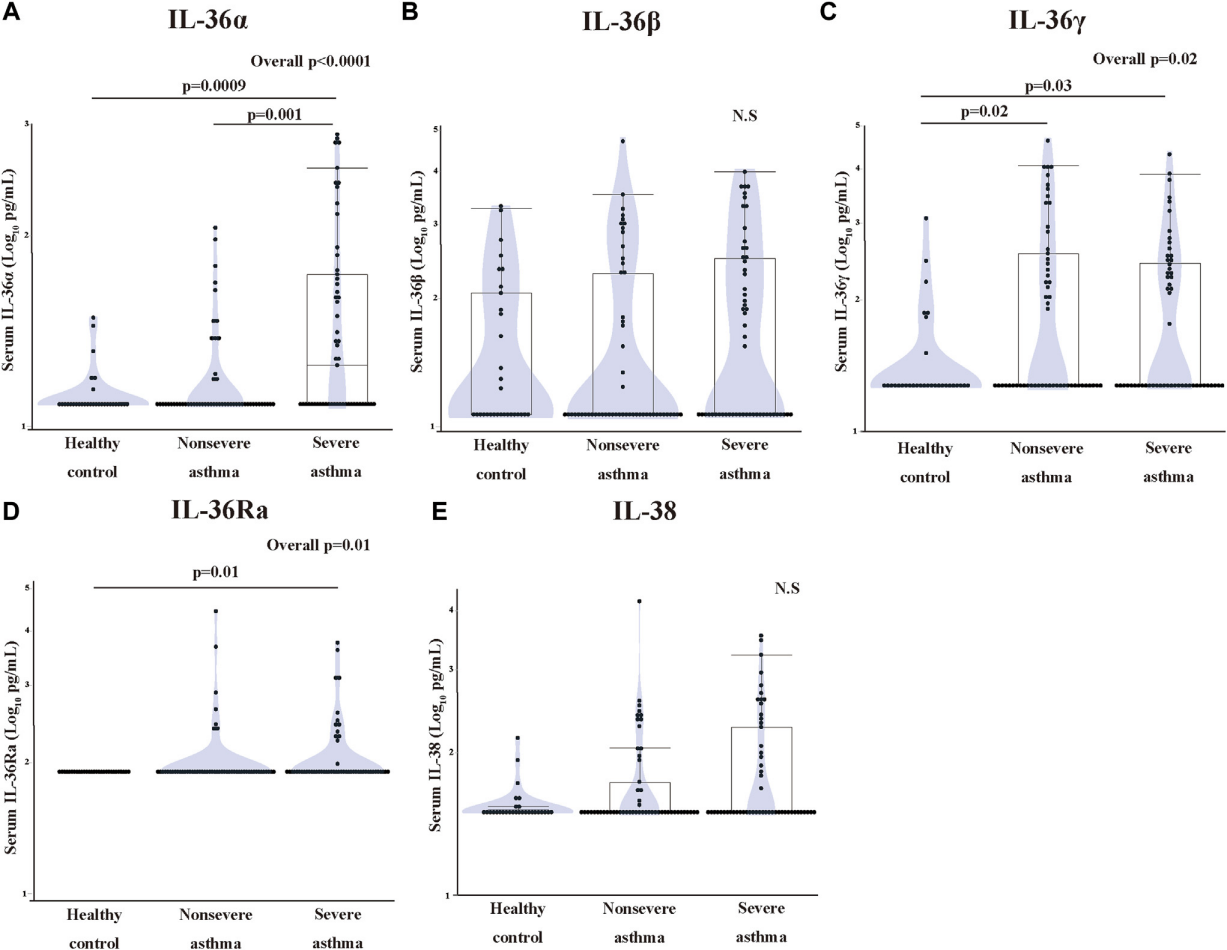
Serum IL-36 subfamily cytokines of patients with nonsevere and severe asthma and healthy controls. Median (interquartile range) (pg/mL) in 31 healthy controls, 55 patients with nonsevere asthma, and 55 patients with severe asthma, respectively, for: **(A)** serum IL-36α: 12.5 (12.5-12.5), 18.3 (12.5-57.4), 12.5 (12.5-12.5), respectively; **(B)** serum IL-36β: 12.5 (12.5-229.0), 12.5 (12.5-365.9), 12.5 (12.5-132.7), **(C)** serum IL-36γ: 18.8 (18.8-362.2), 18.8 (18.8-270.8), 18.8 (18.8-18.8), **(D)** serum IL-36Ra: 93.8 (93.8-93.8), 93.8 (93.8-93.8), 93.8 (93.8-93.8), and **(E)** serum IL-38: 31.2 (31.2-54.1), 31.2 (31.2-183.1), 31.2 (31.2-34.5). Values are log transformed for statistical analysis by Mann-Whitney *U* test and Kruskal-Wallis tests. *Healthy* indicates healthy control participants; *nonsevere,* patients with nonsevere asthma; and *severe,* patients with severe asthma.

**Supplement Fig E1:**
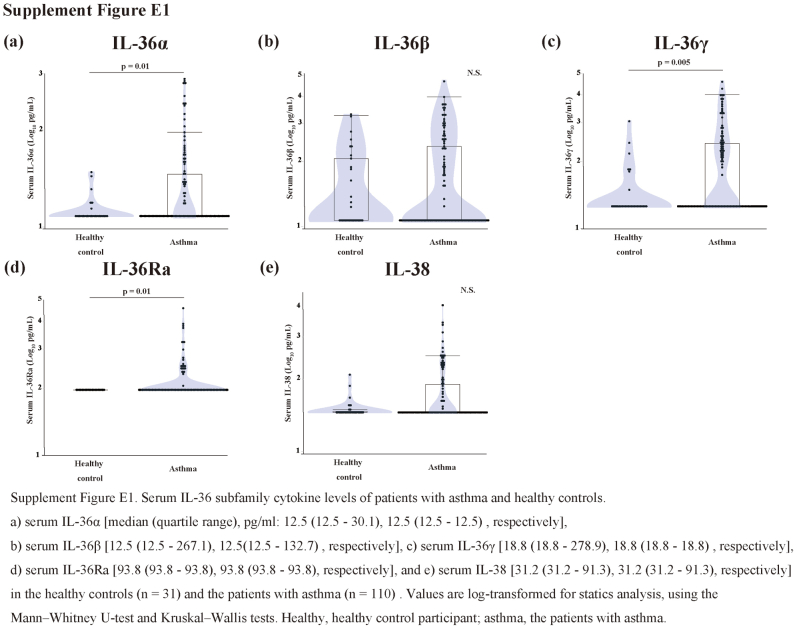


Serum IL-36α, IL-36β, IL-36γ, IL-36Ra, and IL-38 were detected in 37.3%, 42.7%, 43.6%, 18.2%, and 35.5% of asthma patients and in 19.4%, 48.4%, 22.6%, 0, and 22.6% of healthy controls, respectively. The serum IL-36α, IL-36γ, and IL-36Ra levels, but not the IL-36β and IL-38 levels, were significantly higher in asthma patients than healthy controls (*P* = .01, .005, and .01, respectively) (see Fig E1 in the Online Repository available at www.jaci-global.org). Additionally, serum IL-36α levels were significantly higher in severe asthma patients compared to nonsevere asthma patients and healthy controls (median [interquartile range], pg/mL: 18.3 [12.5-57.4], 12.5 [12.5-12.5], and 12.5 [12.5-12.5]; severe vs nonsevere, *P* = .001; and severe vs controls, *P* = .0009, respectively) (Fig 1, *A*). IL-36γ levels were significantly higher in nonsevere and severe asthma patients compared to healthy controls (18.8 [18.8-362.2], 18.8 [18.8-270.8], and 18.8 [18.8-18.8]; nonsevere vs controls, *P* = .02 and severe vs controls, *P* = .03, respectively) (Fig 1, *C*). IL-36Ra levels were significantly higher in severe asthma patients than healthy controls (93.8 [93.8-93.8] and 93.8 [93.8-93.8], *P* = .01, respectively) (Fig 1, *D*). The serum IL-36β and IL-38 levels were comparable between nonsevere and severe asthma patients and healthy controls (Fig 1, *B* and *E*).

### Difference in characteristics between subgroups with low and high IL-36 cytokines

The limited amount of serum IL-36 cytokines detected in asthma patients (Fig 1 and Fig E1) prompted us to determine whether these patients had any asthma manifestations. The detectable lower limits of serum IL-36 cytokines were defined as the reference values of IL-36 to stratify the patients into 2 subgroups according to the distribution of these values in healthy volunteers (Fig 1).

The high IL-36α subgroup exhibited a significantly higher severe asthma rate than the low IL-36α subgroup (*P* = .003). The high IL-36β subgroup was significantly younger and had significantly higher serum IL-6 and IL-17 levels than the low IL-36β subgroup (*P* = .008 and 0.001, respectively). The high IL-36γ subgroup exhibited significantly lower ACT scores and higher serum IL-13 and IL-17 levels than the low IL-36γ subgroup (*P* = .001, .009, and .009, respectively) (Table II).

**Table II:** Characteristics of asthma patients with low and high serum IL-36 subfamily cytokine levels

Characteristic	IL-36α			IL-36β			IL-36γ			IL-36Ra			IL-38		
	Low	High	*P*	Low	High	*P*	Low	High	*P*	Low	High	*P*	Low	High	*P*
No. of participants	69	41		63	47		62	48		90	20		71	39	
Age (years)	64.0 (49.0-72.0)	60.0 (48.5-70.0)	NS	**65.0 (58.0-73.0)**	**56.0 (41.0-71.0)**	**.008**∗	64.0 (53.7-73.0)	60.5 (41.2-71.0)	NS	64.0 (53.7-72.0)	49.5 (36.2-64.7)	NS	65.0 (58.0-72.0)	55.0 (39.0-68.0)	NS
Sex, no. (%)			NS			NS			NS			NS			NS
Female	40 (58.0)	22 (53.6)		35 (55.6)	27 (57.4)		36 (58.1)	26 (54.2)		53 (58.9)	9 (45.0)		40 (56.3)	22 (56.4)	
Male	29 (42.0)	19 (46.3)		28 (44.4)	20 (42.6)		26 (41.9)	22 (45.8)		37 (41.1)	11 (55.0)		31 (43.7)	17 (43.6)	
Smoking history, no. (%)			NS			NS			NS			NS			NS
None	38 (55.2)	19 (46.4)		32 (49.2)	25 (53.2)		28 (45.2)	29 (60.4)		45 (50.0)	12 (60.0)		36 (50.7)	21 (53.9)	
Past	30 (43.4)	21 (51.2)		31 (50.8)	20 (42.6)		32 (51.6)	19		43 (47.8)	8 (40.0)		35 (49.3)	16 (41.0)	
Present	1 (1.4)	1 (2.4)		0	2 (4.2)		2 (3.2)	(39.6)0		2 (2.2)	0		0	2 (5.1)	
Body mass index (kg/m^2^)	23.7 (3.6)	22.7 (3.5)	NS	23.0 (3.3)	23.8 (3.8)	NS	23.0 (3.5)	23.8 (3.6)	NS	23.0 (3.2)	24.7 (4.7)	NS	22.9 (3.2)	24.2 (4.0)	NS
Duration of asthma (years)	9.0 (2.4-16.0)	12.0 (3.0-21.5)	NS	8.0 (2.0-17.0)	12.0 (5.0-20.0)	NS	9.0 (2.0-17.5)	11.0 (3.2-18.5)	NS	10.0 (2.0-17.5)	10.0 (2.0-18.5)	NS	8.0 (2.0-19.0)	12.0 (5.0-17.0)	NS
Allergic rhinitis, no. (%)	42 (60.8)	24 (58.5)	NS	39 (62.0)	27 (57.5)	NS	40 (64.5)	26 (54.2)	NS	55 (61.1)	11 (55.0)	NS	44 (62.0)	22 (56.4)	NS
Chronic sinusitis, no. (%)	19 (27.5)	11 (26.8)	NS	21 (33.3)	9 (19.2)	NS	19 (30.6)	11 (22.9)	NS	28 (31.1)	2 (10.0)	NS	22 (31.0)	8 (20.5)	NS
Medication			.												
ICS dose (μg/d)	400 (400-800)	800 (400-800)	NS	500 (400-800)	500 (400-800)	NS	500 (400-800)	400 (200-800)	NS	450 (400-800)	775 (200-800)	NS	500 (400-800)	500 (400-800)	NS
OCS, no. (%)	8 (11.5)	2 (4.8)	NS	4 (6.3)	6 (12.8)	NS	5 (8.1)	5 (10.4)	NS	7 (7.8)	3 (15.0)	NS	4 (5.6)	6 (15.4)	NS
OCS (mg/d), median (min-max)	0 (0-5)	0 (0-10)	NS	0 (0-10)	0 (0-4)	NS	0 (0-10)	0 (0-5)	NS	0 (0-5)	0 (0-10)	NS	0 (0-10)	0 (0-4)	NS
Biologics†	8 (11.5)	4 (9.7)	NS	8 (12.7)	4 (8.5)	NS	7 (11.2)	5 (10.4)	NS	**12 (13.3)**	**0**	**.02**∗	8 (11.2)	4 (10.2)	NS
Asthma severity			**.003**∗			NS			NS			NS			
Nonsevere	**42 (60.9)**	**13 (31.7)**		35 (55.7)	20 (42.6)		31 (50.0)	24 (50.0)		48 (53.3)	7 (35.0)		37 (52.1)	18 (46.1)	NS
Severe	**27 (39.1)**	**28 (68.3)**		28 (44.3)	27 (57.4)		31 (50.0)	24 (50.0)		42 (46.7)	13 (65.0)		34 (47.9)	21 (53.9)	
ACT (score)	23 (20-25)	24 (19.5-25)	NS	24 (20-25)	23 (19-25)	NS	**24 (21-25)**	**22.5 (18-25)**	**.01**∗	24 (21-25)	21.5 (15.2-25)	NS	**24 (21-25)**	**23 (18-25)**	**.02**∗
Pulmonary function															
FEV_1_ (L)	2.03 (0.79)	2.15 (0.8)	NS	1.96 (0.61)	2.23 (0.9)	NS	2.02 (0.69)	2.15 (0.83)	NS	**1.99 (0.66)**	**2.47 (1.0)**	**.03**∗	1.98 (0.65)	2.25 (0.9)	NS
FEV_1_ (%)	84.2 (18.5)	82.0 (19.6)	NS	83.2 (17.9)	83.7 (20.2)	NS	84.9 (20.1)	81.4 (17.1)	NS	83.5 (19.1)	83.1 (18.0)	NS	83.5 (19.0)	83.1 (18.7)	NS
FVC (L)	2.72 (0.84)	2.85 (0.91)	NS	2.65 (0.73)	2.94 (1.01)	NS	2.65 (0.79)	2.92 (0.95)	NS	**2.67 (0.78)**	**3.23 (1.07)**	**.03**∗	2.68 (0.78)	2.94 (1.0)	NS
FVC (%)	91.1 (14.5)	89.3 (17.1)	NS	89.4 (15.6)	91.9 (15.3)	NS	90.1 (17.0)	90.9 (13.3)	NS	89.9 (15.5)	92.6 (15.5)	NS	90.1 (14.9)	91.0 (16.6)	NS
FEV_1_/FVC (%)	74.1 (10.6)	75.0 (10.0)	NS	73.9 (9.37)	75.1 (11.7)	NS	75.3 (9.36)	73.2 (11.6)	NS	74.1 (9.81)	75.8 (12.9)	NS	73.6 (9.43)	76.0 (12.0)	NS
Blood eosinophil counts (/μL)	188 (120-482)	230 (128-470)	NS	211 (102-381)	228 (130-572)	NS	233 (127-473)	193 (121-487)	NS	215 (121-473)	222 (128-542)	NS	219 (110-453)	196 (128-498)	NS
Blood neutrophil counts (/μL)	3,723 (2,916-5,035)	3,488 (2,677-5,270)	NS	3,674 (2,911-500)	3,625 (2,869-4,702)	NS	3,622 (2,729-5,013)	3,729 (2,959-5,505)	NS	3,601 (2,847-5,166)	4,000 (2,938-5,435)	NS	3,545 (2,858-5,419)	3,729 (2,908-4,847)	NS
Total IgE (IU/L)	181 (66.7-680)	303 (76.5-782)	NS	195 (60-771)	309 (79-623)	NS	188 (71.7-758)	306 (67.2-759)	NS	203 (68.5-786)	312 (87.5-588)	NS	231 (66.0-827)	253 (79.0-486)	NS
Feno (ppb)	25 (16-46)	25 (16-55)	NS	27 (19-38)	24 (15-64)	NS	27 (15-47)	23 (17-48)	NS	24 (15-46)	29 (17-76)	NS	26 (18-46)	25 (15-52)	NS
Serum cytokines (pg/mL)‡															
IL-4	NA	NA	NA	NA	NA	NA	NA	NA	NA	NA	NA	NA	NA	NA	NA
IL-5	NA	NA	NA	NA	NA	NA	NA	NA	NA	NA	NA	NA	NA	NA	NA
IL-6	0.74 (0.48-2.3)	0.98 (0.52-2.31)	NS	0.74 (0.48-1.81)	1.33 (0.48-2.97)	NS	0.74 (0.48-1.97)	1.13 (0.48-2.53)	NS	**0.68 (0.68-1.82)**	**1.91 (1.23-4.31)**	**.004**∗	**0.74 (0.48-1.67)**	**1.72 (0.52-3.42)**	**.02**∗
IL-8	10.5 (7.68-14.7)	12.3 (8.89-26.9)	NS	11.4 (8.12-18.1)	9.92 (7.3-19.3)	NS	11.4 (7.77-17.8)	10.2 (7.83-20.8)	NS	11.4 (7.78-19.1)	9.86 (7.3-15.0)	NS	11.4 (8.12-18.1)	9.86 (7.3-19.3)	NS
IL-10	0.78 (0.78-1.47)	0.78 (0.78-1.68)	NS	0.78 (0.78-1.47)	0.78 (0.78-1.68)	NS	0.78 (0.78-1.01)	0.78 (0.78-1.93)	NS	0.78 (0.78-1.47)	0.78 (0.78-1.9)	NS	0.78 (0.78-1.47)	0.78 (0.78-1.9)	NS
IL-13	0.33 (0.33-0.75)	0.33 (0.33-1.70)	NS	0.33 (0.33-0.75)	0.33 (0.33-1.31)	NS	**0.33 (0.15-0.53)**	**0.33 (0.33-1.65)**	**.009**∗	**0.33 (0.33-0.57)**	**0.46 (0.33-2.68)**	**.01**∗	**0.33 (0.33-0.33)**	**0.46 (0.33-1.81)**	**.001**∗
IL-17	15.6 (15.6-126.3)	15.6 (15.6-244.2)	NS	**15.6 (15.6-15.6)**	**222.8 (41.6-787.8)**	**.001**∗	**15.6 (15.6-73.3)**	**15.6 (15.6-416.2)**	**.009**∗	**15.6 (15.6-76.7)**	**482.9 (22.1-4767)**	**.001**∗	**15.6 (15.6-15.6)**	**278.1 (48.6-1340)**	.**001**∗
TNF	10.3 (5.83-19.4)	10.3 (7.7-18.8)	NS	9.8 (5.31-20.9)	12.0 (6.8-18.1)	NS	9.8 (5.69-17.0)	12.7 (6.73-22.8)	NS	10.3 (6.18-21.2)	10.1 (5.31-16.4)	NS	9.96 (5.69-19.6)	10.9 (6.18-19.6)	NS
IFN-γ	1.21 (0.8-1.86)	1.86 (1.34-3.12)	NS	1.35 (0.92-2.45)	1.35 (0.8-2.12)	NS	1.34 (0.8-2.12)	1.48 (1.07-2.39)	NS	**1.34 (0.8-2.12)**	**1.86 (1.34-3.77)**	**.02**∗	1.34 (0.92-2.30)	1.66 (0.89-2.27)	NS

Parametric data are expressed as medians (standard deviations) and nonparametric data as medians (25-75%) unless otherwise indicated. Serum IL-4 and IL-5 were not detected. ICS dose values are described as converted to corresponding dose to fluticasone propionate: 2 mg beclomethasone = 2 mg budesonide = 1 mg mometasone = 0.2 mg fluticasone furoate = 1 mg fluticasone propionate. Asthma patients were classified into low or high serum IL-36 group according to lower limit of detection of serum IL-36: those with levels below the lower limit were assigned to low IL-36 group, and those with levels above the lower limit were assigned to high IL-36 group. If serum cytokines values were not detected, then lower limit values were adopted for analysis. Statistically significant values are shown in boldface.

*ICS,* Inhaled corticosteroid; *LABA,* long-acting β-agonist; *LAMA,* long-acting muscarinic antagonist; *LTRA,* leukotriene receptor antagonist; *NA,* not applicable; *NS,* not significant; *OCS,* oral corticosteroids.

†Biologics include 1 omalizumab, 5 mepolizumab, 5 benralizumab, and 1 dupilumab.

∗ Statistically significant.

‡ Serum cytokines were measured with Bio-Plex human 17 cytokine assay kits and Bio-Plex suspension array system, except for IL-17.

The high IL-36Ra subgroup did not receive any therapy with biologics, but they had significantly higher FEV_1_, FVC, and serum IL-6, IL-8, IL-13, IL-17, and IFN-γ levels than the low IL-36Ra subgroup (*P* = .02, .03, .03, .004, .01, .001, and .02, respectively). The high IL-38 subgroup exhibited significantly lower ACT scores and higher serum IL-6, IL-13, and IL-17 levels than the low IL-38 subgroup (*P* = .02, .02, .001, and 0.001, respectively). There were no other differences between the low and high IL-36 subgroups (Table II).

**Fig 2:**
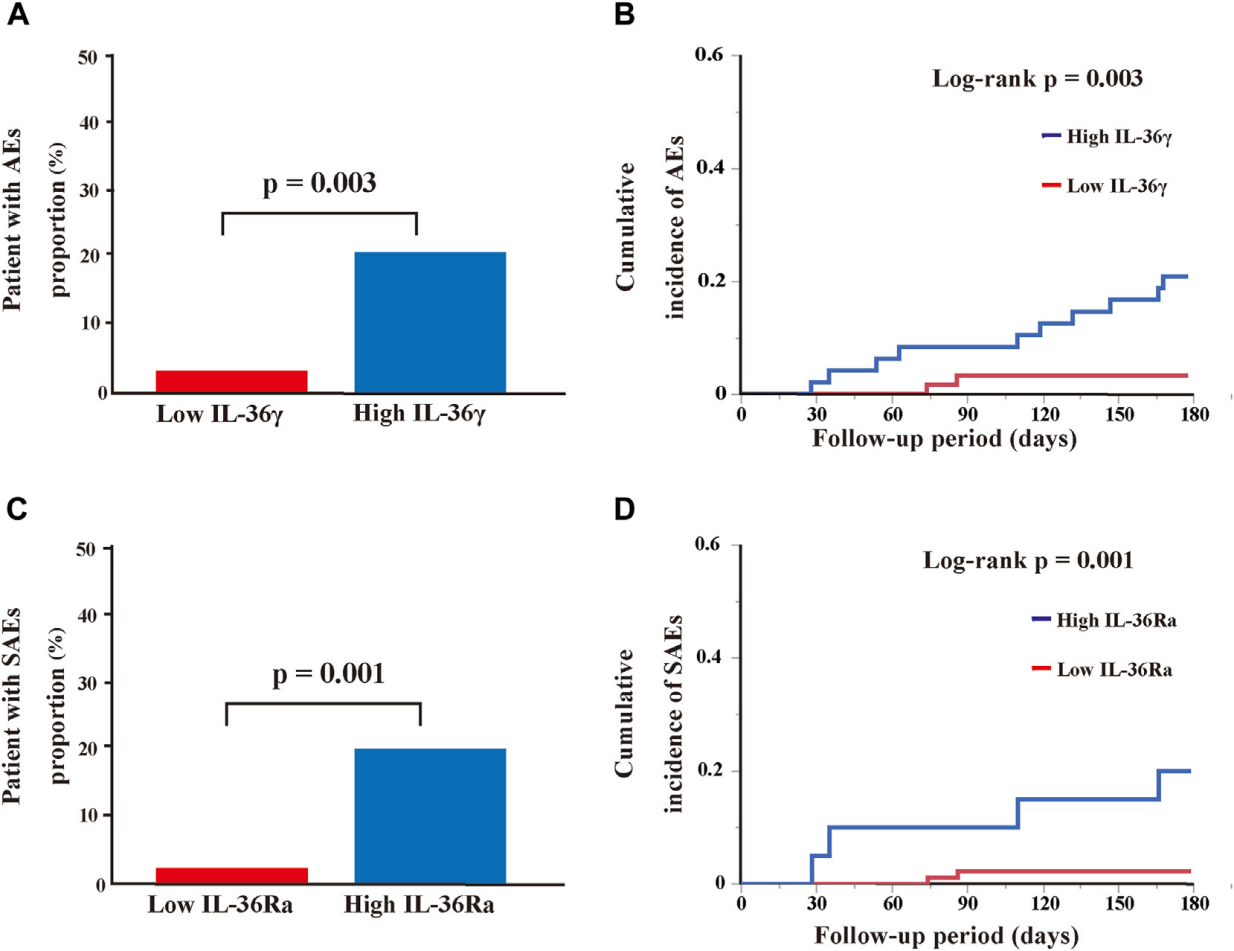
Ratio of patients experiencing AE and time to first AE in 6 months. Comparison of **(A)** ratio of patients having AE and **(B)** free time to first AE between high and low serum IL-36γ subgroups. Comparison of **(C)** ratio of patients with SAE and **(D)** time to first SAE between high and low serum IL-36Ra subgroups.

The proportion of asthma patients with AEs and the number of AEs were significantly higher in the high IL-36γ subgroup than in the low IL-36γ subgroup (both *P* = .003) (Fig 2, *A,* and see Table E1 in the Online Repository available at www.jaci-global.org). The time to first AE was significantly shorter in the high IL-36γ subgroup than in the low IL-36γ subgroup (mean, 164 [95% confidence interval (CI), 153-175] days vs 177 [172-181] days, *P* = .003, Fig 2, *B*). The higher ACT scores and serum IL-13 and IL-17 levels in the high IL-36γ subgroup (Table II) were not associated with HR for subsequent AEs. The HR for subsequent AEs after baseline was significantly higher in the high IL-36γ subgroup than in the low IL-36γ subgroup, after adjusting for age and sex (mean, 6.57 [95% CI, 1.42-30.43], *P* = .016).

The ratio of the asthma patients with SAEs and the number of SAEs were significantly higher in the high IL-36Ra subgroup than in the low IL-36Ra subgroup (both *P* = .001) (Fig 2, *C,* and Table E1). The mean time to first SAE was significantly shorter in the high IL-36Ra subgroup than in the low IL-36Ra subgroup (161 [95% CI, 141-181] days vs 178 [175-181] days, *P* = .001, Fig 2, *D*). The lower biologic receipt rate; higher FEV_1_ and FVC; and higher serum IL-6, IL-13, IL-17, and IFN-γ levels in the high IL-36Ra subgroup (Table II) were not associated with HR for subsequent SAE. The HRs for subsequent SAE were significantly higher in the high IL-36Ra subgroup than in the low IL-36Ra subgroup, after adjusting for age and sex (7.10 [95% CI, 1.15-43.95], *P* = .011).

**Supplement Fig E2:**
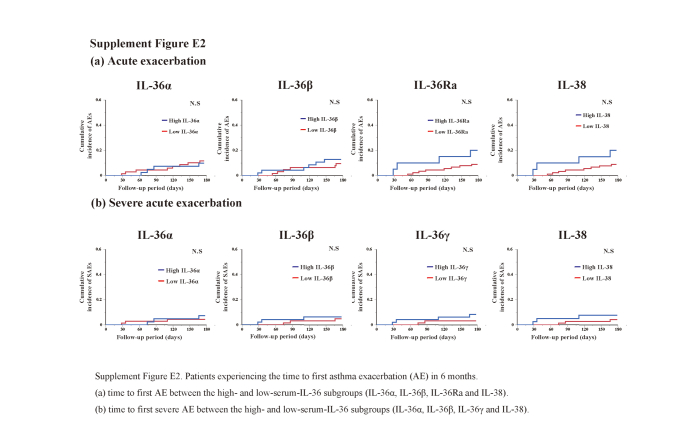


In the other high IL-36 subgroups, the time to the first AE and SAE and HR for subsequent AEs and SAEs did not differ from those of the corresponding low IL-36 subgroups (see Fig E2 in the Online Repository available at www.jaci-global.org).

### Associations of serum IL-36 cytokine levels with clinical characteristics

**Fig 3:**
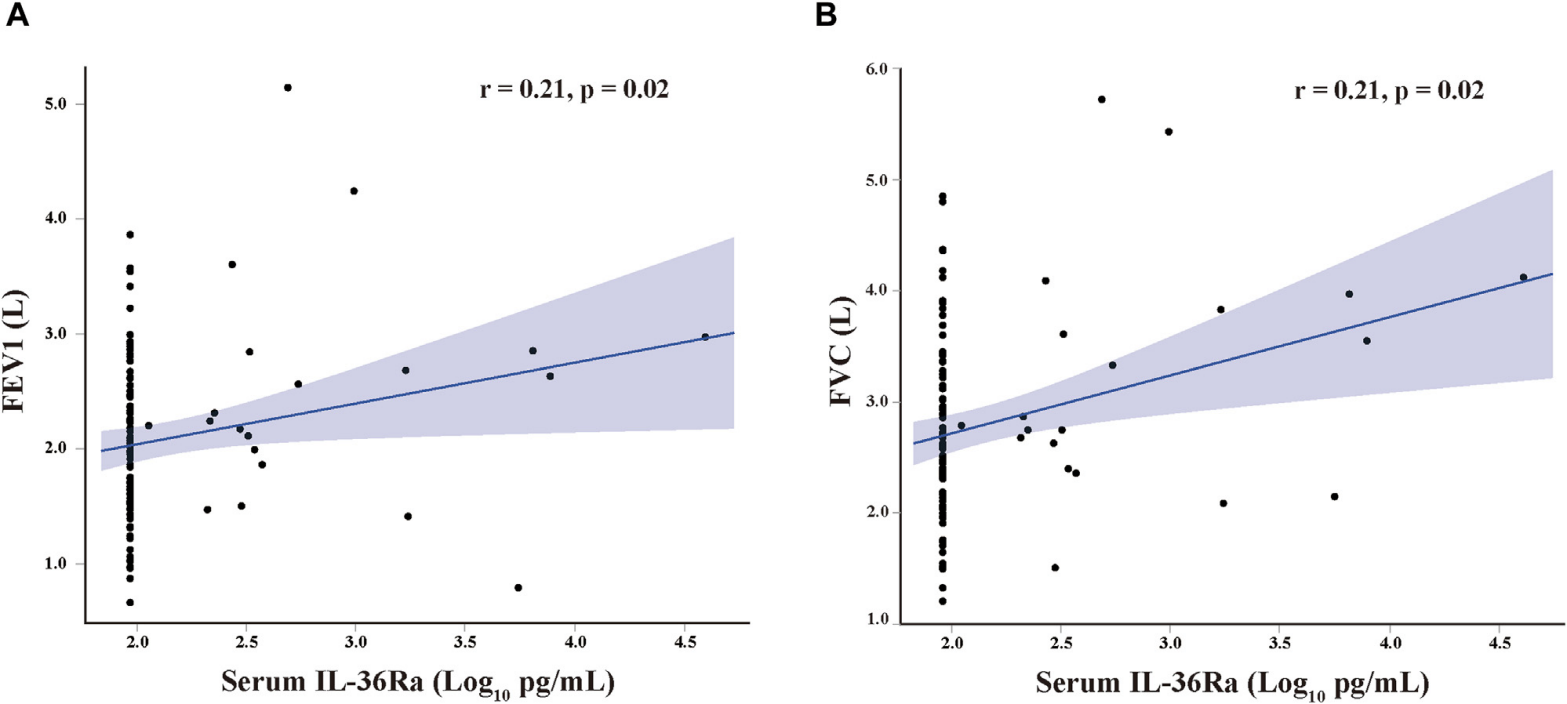
Relationships between serum IL-36Ra and pulmonary function in patients with asthma. Relationships between serum IL-36Ra and **(A)** FEV_1_ (*r* = 0.21, *P* = .02) or **(B)** FVC (*r* = 0.21, *P = .*02) in asthma patients, adjusted by age as covariance. Statistical significance was determined by Spearman correlation test and partial correlation analysis.

The increase in serum IL-36Ra levels was slightly but significantly correlated with increased FEV_1_ (*P* = .02) and FVC (*P* = .02) in all asthma patients after adjusting for age (Fig 3, and see Table E2 in the Online Repository available at www.jaci-global.org), but the other pulmonary function parameters, Feno and ACT score, did not show any correlation. The other serum IL-36 levels were not correlated with any pulmonary function parameters, Feno, or ACT scores in the asthma patients.

### Correlation between serum IL-36 cytokine agonist and antagonist levels

Because elevated serum IL-36Ra levels offered contradictory aspects regarding asthma in this study, we examined the correlations between serum agonistic and antagonistic IL-36 cytokine levels. As expected, the serum IL-36Ra levels were significantly and positively correlated with serum IL-36β, IL-36γ, and IL-38 levels (*P* < .0001 for all), but not with serum IL-36α levels. The serum IL-38, IL-36β, and IL-36γ levels were significantly and positively correlated with each other (Table III).

**Table III:** Correlation among serum IL-36 subfamily cytokine levels in patients with asthma

*r / P*	IL-36α	IL-36β	IL-36γ	IL-36Ra	IL-38
**IL-36α**		.3	.34	.12	.91
**IL-36β**	−0.09		<.0001∗	<.0001∗	<.0001∗
**IL-36γ**	−0.09	**0.37**∗		<.0001∗	.0002∗
**IL-36Ra**	0.14	**0.48**∗	**0.4**∗		<.0001∗
**IL-38**	−0.009	**0.83**∗	**0.34**∗	**0.59**∗	

*P* values were calculated by Spearman correlation test. Statistically significant *r* values are shown in boldface.

∗ Statistically significant.

### Correlation between IL-36 cytokines and serum levels of T2 and non-T2 inflammatory mediators

**Fig 4:**
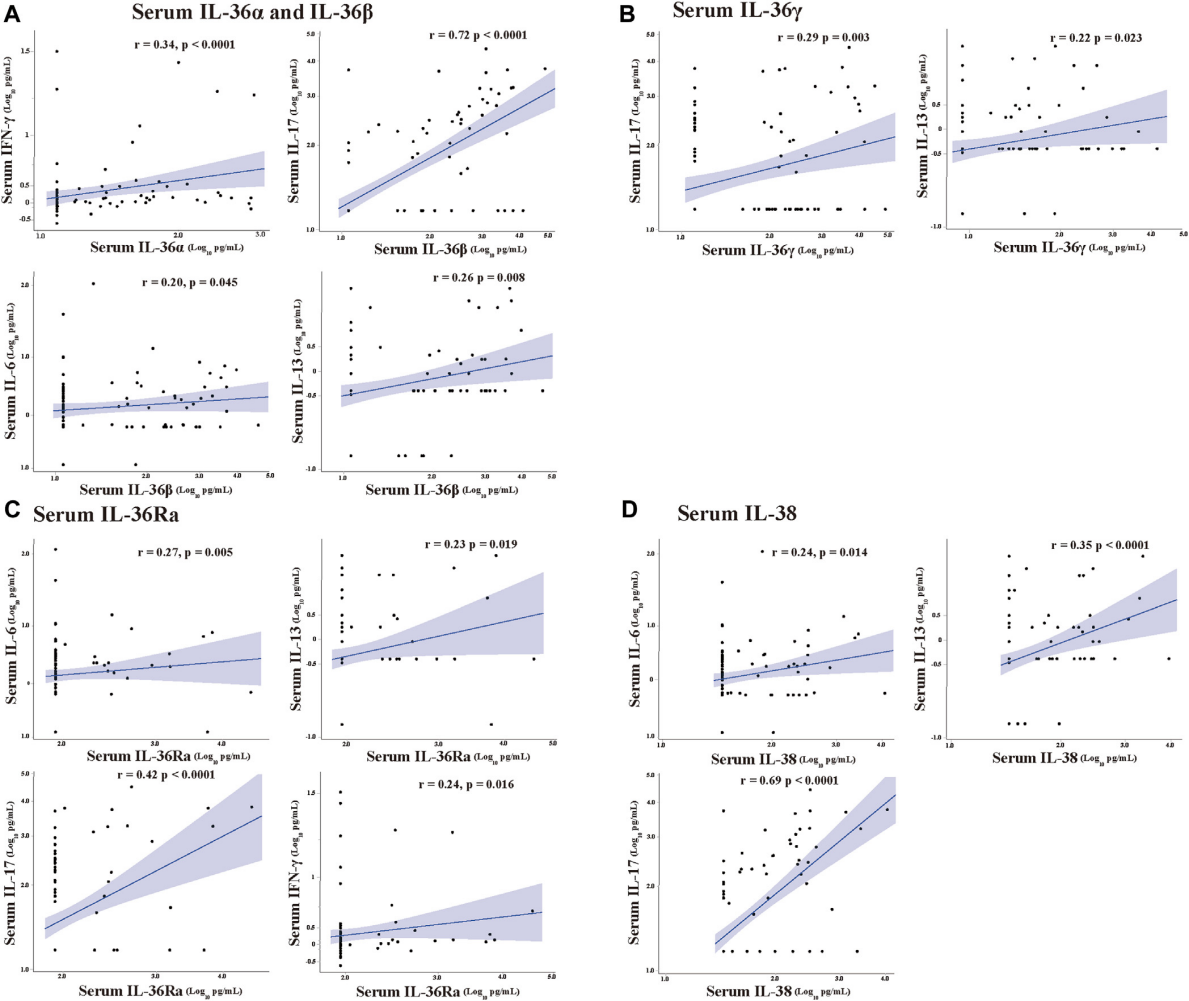
Correlations of serum IL-36 subfamily cytokine and serum cytokines in patients with asthma. Correlation of **(A)** IL-36α with IFN-γ (*r* = 0.34, *P* < .0001); IL-36β with IL-6 (*r* = 0.20, *P = .*045), IL-13 (*r* = 0.26, *P = .*008), and IL-17 (*r* = 0.72, *P* < .0001); **(B)** IL-36γ with IL-13 (*r* = 0.22, *P = .*023) and IL-17 (*r* = 0.29, *P = .*003); **(C)** IL-36Ra with IL-6 (*r* = 0.27, *P = .*005), IL-13 (*r* = 0.23, *P = .*019), IL-17 (*r* = 0.42, *P* < .0001), and IFN-γ (*r* = 0.34, *P = .*016); and **(D)** IL-38 with IL-6 (*r* = 0.24, *P = .*014), IL-13 (*r* = 0.35, *P* < .0001), and IL-17 (*r* = 0.69, *P* < .0001). Statistical significance was determined by Spearman and partial correlation analysis.

We examined the correlations of serum IL-36 levels with serum IL-6, IL-8, IL-10, IL-13, IL-17, IFN-γ, and TNF levels, except for IL-4 and IL-5 levels, which were only rarely observed in our asthma patients. Each serum IL-36 level was positively and moderately correlated with the specific inflammatory mediators (Fig 4, and see Table E3 in the Online Repository available at www.jaci-global.org): serum IL-36α levels with serum IFN-γ levels (*P* < .0001) (Fig 4, *A*); serum IL-36β levels with serum IL-6 (*P* = .045), IL-13 (*P* = .008), and IL-17 levels (*P* < .0001) (Fig 4, *A*); serum IL-36γ levels with serum IL-13 (*P* = .023) and IL-17 levels (*P* = .003) (Fig 4, *B*); serum IL-36Ra levels with serum IL-6 (*P* = .005), IL-13 (*P* = .019), IL-17 (*P* < .0001), and IFN-γ levels (*P* = .016) (Fig 4, *C*); and serum IL-38 levels with serum IL-6 (*P* = .014), IL-13 (*P* < .0001), and IL-17 levels (*P* < .0001) (Fig 4, *D*), but not with the other cytokines (Table E3).

## Discussion

The present study demonstrated several associations of serum IL-36 cytokines with asthma severity and clinical manifestations, including increased serum IL-36α, IL-36γ, and IL-36Ra levels in patients with asthma, especially severe asthma; the positive correlation of serum IL-36Ra with some pulmonary functions in asthma patients; and the relationship of serum IL-36 cytokines with each other and with serum T2 and non-T2 cytokines among the asthma patients. Furthermore, this study demonstrated that asthma patients with high serum IL-36γ, IL-36Ra, and/or IL-38 had decreased ACT scores, increased frequency of AEs or SAEs, high HR for subsequent AEs or SAEs, and shorter time to first AE or SAE in 6 months. These results in this study are consistent with those in other studies that show an association of IL-36 cytokines with T2 and non-T2 inflammation in respiratory diseases and with the severity of the respiratory diseases.^23^, ^24^, ^25^, ^26^, ^27^, ^28^^,^^35^ Hence, a novel contribution of systemic IL-36 cytokine alteration to the clinical manifestations of asthma has emerged in this study.

We found that several serum IL-36 cytokines exhibited different patterns compared to the sputum IL-36 findings from the study of Li et al,^30^ which showed elevated IL-36β, comparable IL-36γ, and reduced IL-36Ra levels in patients with mild asthma compared to healthy controls. This discrepancy may be attributed to the different cell sources of IL-36 cytokines in the airway versus blood. Reportedly, IL-36 cytokines are secreted by airway epithelial cells and various immune cells, particularly macrophages, in the blood.^12^^,^^13^ In patients with COPD, IL-36α and IL-36γ genes were spontaneously expressed in small airway epithelial cells, with a concomitant decrease in *IL36RN* (IL-36Ra gene) expression detected—a pattern not observed in lung tissue macrophages.^27^ However, peripheral blood monocytes from patients with COPD demonstrated increased IL-36α and IL-36γ expression as disease severity worsened.^24^ Asthma and COPD share characteristics as chronic obstructive airway diseases; in patients with asthma, IL-36 cytokines in the airways are primarily produced by airway epithelial cells, whereas in the blood, monocytes are likely the main source. Additionally, disease severity affects IL-36 cytokine release by specific cells types. In our study, serum IL-36Ra levels in patients with nonsevere asthma were comparable to those in healthy controls, aligning with findings from patients with mild asthma.^30^ Given the heightened activation of immune cells in severe asthma, the differing trends in IL-36γ and IL-36Ra between sputum and blood may be attributed to varying activity in the originating cells and asthma severity, indicating that the specificity of IL-36 cytokines for airway or systemic inflammation could depend on their isoforms and on disease severity.

Systemic IL-36α exhibited distinct behavior compared to IL-36β and IL-36γ in our study: serum IL-36α levels showed no correlation with other IL-36 cytokines or clinical indices. These results contradict findings in lung tissue, in which CXCL8 production by IL-36α–stimulated macrophages in patients with COPD was similar to that induced by IL-36β– and IL-36γ–stimulated macrophages.^24^^,^^27^ IL-36α may play a more prominent role in airway immunopathology compared to systemic circulation, as sputum IL-36α levels were elevated and strongly correlated with IL-6 in patients with mild asthma.^30^

Asthma patients with high IL-36γ levels had a deteriorated condition of asthma control, indicated by lower ACT scores, more frequent AEs, and a shorter time to the first AE after study entry. Our findings are consistent with the findings of several studies showing that asthma patients with systemically elevated IL-6 levels exhibited reduced pulmonary function, low ACT, and more frequent AEs, SAEs, and hospitalizations^36^^,^^37^ and that the increased expression of IL-17 in the bronchial mucosa was correlated with increased SAEs and decline in percentage predicted FEV_1_.^38^ Indeed, the IL-6 and IL-17 levels were increased in the high IL-36γ subgroup and positively correlated with IL-36γ in the asthma patients in our study. Therefore, systemic IL-36γ, similar to IL-6 and IL-17 levels, may affect the asthmatic condition. Additionally, the elevated IL-36α levels in severe asthma patients in this study support the agonistic effects of IL-36 cytokines on the advanced asthma condition. Systemic agonistic IL-36 cytokines, especially IL-36γ, may promote systemic inflammation among asthma patients, leading to worsening of the asthma condition.

IL-36β, IL-36γ, IL-36Ra, and IL-38 were also positively correlated with IL-13 in our asthma patients. Consistent with our findings, a previous study has reported that the mRNA expression and protein of these IL-36 cytokines were upregulated in the nasal tissues of eosinophil-dominant chronic rhinosinusitis,^28^ that peripheral blood eosinophils were activated by IL-36γ in allergic rhinitis patients,^23^ and that T2 airway inflammation and cytokine production was reduced by IL-36Ra and IL-38 in causative allergen-exposed mice,^17^^,^^18^^,^^27^ suggesting that the IL-36 cascade promotes allergic airway inflammation. Therefore, IL-36 cytokines are likely associated with T2 inflammation.

The present study demonstrated an efficient association of IL-36R with pulmonary function among asthma patients. Our findings are consistent with evidence regarding the potential allergic anti-inflammatory effects of IL-36Ra and IL-38.^17^, ^18^, ^19^^,^^27^ IL-36Ra reduced house dust mite (HDM)- and ovalbumin-induced allergic and nonallergic airway inflammation in allergen-sensitized mice.^17^^,^^19^ IL-38 inhibited the HDM-induced infiltration of the T2 and non-T2 immune cells and cytokines in the lungs of HDM-sensitized mice.^17^^,^^18^ Additionally, IL36RN (IL-36 receptor antagonist) attenuated the production of non-T2 cytokines by lipopolysaccharide-stimulated mononuclear cells among children with moderate asthma.^19^ Thus, this study convincingly suggests that antagonistic IL-36 cytokines exhibit protective effects on T2 and non-T2 lung inflammation to preserve lung function in asthma. The IL-36 receptor may be a new therapeutic target for uncontrolled asthma patients with elevated serum IL-36 levels, as the anti–IL-36R monoclonal antibody can be administered to patients with generalized pustular psoriasis in Japan.^39^^,^^40^

In the present study, asthma patients with high IL-36Ra and IL-38 levels showed a deteriorating asthma status and were prone to SAEs; additionally, IL-36Ra and IL-38 were positively correlated with the agonistic IL-36 cytokines IL-6, IL-13, IL-17, and IFN-γ. Considering the anti-inflammatory effect of IL-36Ra, IL-38, and *IL36RN* reported in several studies,^17^, ^18^, ^19^^,^^27^^,^^39^^,^^40^ these findings suggest that the level of systemic antagonistic IL-36 cytokines increases to counteract the effect of proinflammatory cytokines and the worsening asthma condition and to prevent the development of future complications. Thus, the high levels of serum antagonistic IL-36 cytokines, especially IL-36Ra, may be counterbiomarkers of the current unstable asthma status and future AEs.

A limitation of this study is that we utilized the low detection limit of IL-36 cytokines to categorize asthma patients into high and low IL-36 subgroups. This approach may have underestimated the number of patients in the high/low IL-36 subgroups, and the results may be affected by potential selection bias. However, most patients in the high IL-36 subgroup were above the 75th percentile for IL-36 cytokines among all asthma patients in this study, indicating that the high IL-36 subgroup was sufficiently heterogeneous. Our cutoff values revealed distinct characteristics of asthma patients with high IL-36 cytokine levels, suggesting that IL-36 could be a favorable treatment target. Further research is needed to establish more reliable cutoff values for serum IL-36 to better characterize patients with asthma. This study highlights the potential clinical relevance of IL-36 cytokines in asthma management, and we anticipate that a prospective study will verify their roles in asthma.

In conclusion, IL-36 cytokines exhibit different characteristics in asthma patients, including association of IL-36α with asthma severity and of IL-36Ra and IL-38 with better pulmonary function and involvement of IL-36γ, IL-36Ra, and IL-38 in current asthma control and future exacerbations. IL-36 cytokines could affect the clinical manifestations and systemic T2 and non-T2 immune pathways in adults with asthma.

**Clinical implications**

• Systemically high serum IL-36 cytokines are associated with asthma severity, poor asthma control, and increased risk of acute exacerbations.
• This relationship involves both T2 and non-T2 cytokines.

## Disclosure statement

Supported by the 10.13039/501100001691Japan Society for the Promotion of Science (Grant-in-Aid for Scientific Research JP21K08445) and the 10.13039/100020180Japanese Society of Allergology (Basic Research Support Program 2022).

Disclosure of potential conflict of interest: Y. Hoshino, H. Iemura, T. Uchida, Y. Uchida, T. Soma, K. Nakagome, and M. Nagata declare personal fees from 10.13039/100004325AstraZeneca, GSK, Sanofi, and Novartis Pharma. The rest of the authors declare that they have no relevant conflicts of interest.

## Supplementary data

Supplement Tables

Supplementary Method
