# Supplementary material for: Influence of serum IL-36 subfamily cytokines on clinical manifestations of asthma
Source: J Allergy Clin Immunol Glob. 2025 Jan 18;4(2):100419. doi: 10.1016/j.jacig.2025.100419 (PMC11925522; doi:10.1016/j.jacig.2025.100419)
Supplement: Supplement Tables [file mmc1.docx]

**Supplement Table E1. Comparison of acute exacerbation occurrences between patients with asthma and low or high serum IL-36 levels**

|  | **IL-36α** | | | **IL-36β** | | | **IL-36γ** | | | **IL-36Ra** | | | **IL-38** | | |
| --- | --- | --- | --- | --- | --- | --- | --- | --- | --- | --- | --- | --- | --- | --- | --- |
|  | **Low**  **N = 69** | **High**  **N = 41** | ***P* value** | **Low**  **N = 63** | **High**  **N = 47** | ***P* value** | **Low**  **N = 62** | **High**  **N = 48** | ***P* value** | **Low**  **N = 90** | **High**  **N = 20** | ***P* value** | **Low**  **N = 71** | **High**  **N = 39** | ***P* value** |
| **Patients with AEs, n (%)** | 8 (11.5) | 4 (9.7) | N.S. | 6 (9.5) | 6 (12.7) | N.S. | **2 (3.2)** | **10 (20.8)** | **0.003** | 8 (8.8) | 4 (20.0) | N.S. | 6 (8.5) | 6 (15.4) | N.S. |
| **Number of AEs,**  **n/person, median (min**–**max)** | 0.0  (0.0–5.0) | 0.0  (0.0–1.0) | N.S. | 0.0  (0.0–1.0) | 0.0  (0.0–5.0) | N.S. | **0.0**  **(0.0**–**1.0)** | **0.0**  **(0.0**–**5.0)** | **0.003** | 0.0  (0.0–1.0) | 0.0  (0.0–5.0) | N.S. | 0.0  (0.0–1.0) | 0.0  (0.0–5.0) | N.S. |
| **Patients with severe AEs, n (%)** | 3 (4.3) | 3 (7.3) | N.S. | 3 (4.7) | 3 (6.3) | N.S. | 2 (3.2) | 4 (8.3) | N.S. | **2 (2.2)** | **4 (20.0)** | **0.001** | 3 (4.2) | 3 (7.6) | N.S. |
| **Number of severe AEs,**  **n/person, median (min–max)** | 0.0  (0.0–5.0) | 0.0  (0.0–1.0) | N.S. | 0.0  (0.0–1.0) | 0.0  (0.0–5.0) | N.S. | 0.0  (0.0–1.0) | 0.0  (0.0–5.0) | N.S. | **0.0**  **(0.0**–**1.0)** | **0.0**  **(0.0**–**5.0)** | **0.001** | 0.0  (0.0–1.0) | 0.0  (0.0–5.0) | N.S. |

Patients with asthma were classified into low and high serum IL-36 groups per the lower limit of serum IL-36 detection: below the limit = low IL-36 group; above the limit = high IL-36 group. Significant p values are shown in bold. Data are expressed as medians (min–max). AE, asthma exacerbation.

**Supplement Table E2. Correlations between serum IL-36 subfamily cytokine levels and clinical indices in patients with asthma**

|  | **IL-36α** | **IL-36β** | **IL-36γ** | **IL-36Ra** | **IL-38** |
| --- | --- | --- | --- | --- | --- |
| **FEV1** | 0.1 | 0.13 | 0.09 | **0.21*** | 0.14 |
| **%FEV1** | −0.05 | −0.03 | −0.08 | −0.02 | −0.07 |
| **FVC** | 0.12 | 0.13 | 0.18 | **0.21*** | 0.11 |
| **%FVC** | 0.01 | 0.04 | 0.05 | 0.04 | −0.06 |
| **FEV1/FVC** | −0.08 | 0.06 | −0.13 | 0.07 | 0.1 |
| **FeNO** | −0.05 | −0.009 | −0.06 | 0.09 | 0.01 |
| **ACT** | 0.08 | 0.16 | −0.19 | −0.14 | −0.001 |

R values are provided. P values were calculated using Spearman’s correlation test, with significant p values shown in bold. Coefficient values for correlations between serum IL-36 subfamily cytokines and FEV1 or FVC were evaluated using partial correlation analysis, with age included as a covariate when the correlations were significant.

FEV1, fractional expiratory volume in 1 s; FVC, forced vital capacity

*: p = 0.02

**Supplement Table E3. Correlations between IL-36 subfamily cytokine levels and type 2 and nontype 2 inflammatory mediator levels in serum**

|  | **IL-36α** | **IL-36β** | **IL-36γ** | **IL-36Ra** | **IL-38** |
| --- | --- | --- | --- | --- | --- |
| **IL-4** | N. A. | N. A. | N. A. | N. A. | N. A. |
| **IL-5** | N. A. | N. A. | N. A. | N. A. | N. A. |
| **IL-6** | 0.1 | **0.20†** | 0.06 | **0.27¶** | **0.24††** |
| **IL-8** | 0.12 | −0.08 | −0.07 | −0.06 | −0.07 |
| **IL-10** | 0.01 | 0.09 | 0.11 | 0.06 | 0.11 |
| **IL-13** | 0.11 | **0.26‡** | **0.22§** | **0.23#** | **0.35*** |
| **IL-17** | −0.001 | **0.72*** | **0.29‖‖** | **0.42*** | **0.69*** |
| **IFN-γ** | **0.34*** | −0.004 | 0.16 | **0.24**** | 0.1 |
| **TNF** | 0.09 | 0.09 | 0.12 | −0.03 | 0.07 |

R values are provided. P values were calculated using Spearman’s correlation test, with significant p values shown in bold. Correlations between IL36 cytokine levels and IL-4 and IL-5 levels are not shown, as IL-4 and IL-5 were rarely detected. *: p < 0.0001, †: p = 0.045, ‡: p = 0.008, §: p = 0.023, ‖‖: p = 0.003, ¶: p = 0.005, #: p = 0.019, **: p = 0.016, ††: p = 0.014; N.A. not applicable
