# Supplementary material for: Influence of serum IL-36 subfamily cytokines on clinical manifestations of asthma
Source: J Allergy Clin Immunol Glob. 2025 Jan 18;4(2):100419. doi: 10.1016/j.jacig.2025.100419 (PMC11925522; doi:10.1016/j.jacig.2025.100419)
Supplement: Supplementary Method [file mmc2.docx]

**Supplementary Methods**

***Patients and study design***

This single-center, cross-sectional, observational study involved patients with asthma and healthy volunteers aged >20 years, recruited from the Allergy Centre at Saitama Medical University Hospital between March 2020 and December 2023. Asthma was diagnosed per the Japanese Society of Allergology guidelines 2017 (#E1). Briefly, patients with asthma typically had asthmatic symptoms or exacerbations and presented a positive bronchial reversibility as reflected by bronchodilator use or airway hyper-responsiveness (PC20 methacholine < 8 mg/mL). Severe asthma was defined according to the American Thoracic/European Respiratory Society (ATS/ERS) guidelines (#E2), referencing the use of high-dose inhaled corticosteroids [fluticasone propionate (FP) or other agents equivalent to FP ≥ 1,000 µg], combined with a long-acting β-agonist, leukotriene modifier, or slow-release theophylline modifier over the previous year of the study, or oral corticosteroids for >6 months of the previous year of the study, or any biologic therapy for >3 months. Patients were excluded if they had severe asthma exacerbations (SAEs) or respiratory infections within 4 weeks before the study, other lung diseases (such as allergic pulmonary aspergillosis and eosinophilic granulomatosis with polyangiitis), severe comorbidities (such as malignancy, severe renal failure, or severe heart failure), or were pregnant. All participants underwent blood tests, pulmonary function tests, and measurement of the fraction of exhaled nitric oxide (FeNO) and were asked to complete the asthma control test. Written informed consent was obtained from all participants before their inclusion. The study received ethical approval from the Institutional Review Board of Saitama Medical University Hospital (approval number: 19132.02) and was also registered in the UMIN Clinical Trials Registry (UMIN 000049253).

***Measurement of serum IL-36 subfamily cytokines and other cytokines***

Serum levels of IL-36α, IL-36β, IL-36γ, IL-36Ra, and IL-38 were measured using enzyme-linked immunosorbent assay (ELISA) kits (R&D Systems, Minneapolis, USA) per the manufacturer’s instructions. The detection limits were 12.5, 12.5, 18.8, 93.8, and 31.2 pg/mL for IL-36α, IL-36β, IL-36γ, IL-36Ra, and IL-38, respectively. Serum Il-4, -5, -6, -8, -13, -17, IFN-γ, and TNF concentrations were measured using ELISA (R&D Systems) or Bio-Plex Human Cytokine 17 assay kits with the Bio-Plex Suspension Array System (Bio-Rad, Mississauga, Canada), per the manufacturer’s instructions. If any values were below the detection limits, the lower limit provided by the kits was used in the analysis.

***Pulmonary function test and FeNO***

Pulmonary function tests were conducted using an AS307 spirometer (Minato Medical Science, Osaka, Japan) (#E3). FeNO levels were measured using a compact device (NIOX VERO, Circassia AB, Oxford, United Kingdom) according to ATS recommendations (#E4).

***Stratification between patients with and without elevated serum IL-36 subfamily cytokine levels***

To differentiate patients with asthma and elevated serum IL-36 subfamily cytokine levels from those without such elevation, we used the detection limits of these cytokines as reference values. The distribution of these values in healthy volunteers (Figure 1) indicated that a subset of patients with asthma had detectable serum IL-36 cytokine levels, which was distinct from those without detectable levels. Therefore, patients with asthma were categorized into two subgroups: those with low (lo-IL-36) and high (hi-IL-36) serum IL-36 levels. Next, we compared various clinical indices, pulmonary function, FeNO, serum cytokine levels, number of AEs within 6 months after baseline, time to first AE during the 6 months after baseline, and hazard ratio (HR) for subsequent AEs between the two groups. An SAE was defined according to ATS/ERS guidelines (#E5) as the need for systemic corticosteroids for ≥2 consecutive days or hospitalization. An AE was defined as the use of a rescue bronchodilator for ≥2 consecutive days because of the worsening symptoms or lung function or as the occurrence of an SAE.

***Data analysis***

For statistical analysis, serum IL-36 subfamily cytokine levels were log-transformed. The Shapiro–Wilk test was used to assess the normality of the data distribution. Normally distributed continuous variables were expressed as means ± standard deviations, whereas non-normally distributed data were presented as medians with interquartile ranges. Student’s t-test and analysis of variance were used for normally distributed variables, whereas Mann–Whitney U and Kruskal–Wallis tests were applied to non-normally distributed variables. Categorical variables were compared using the Chi-square test. Spearman’s correlation test was used to determine associations between parameters. Significant correlations between IL-36 subfamily levels and pulmonary function, such as fractional expiratory volume in 1 s (FEV1) or forced vital capacity, were further analyzed using partial correlation analysis, adjusting for age as a covariate. The time to first AE during 6 months after baseline was evaluated using the log-rank test, with data from patients who experienced AEs or were lost to follow-up censored during the analysis. The HRs for subsequent AEs were calculated using Cox proportional hazards modeling and adjusted for age, sex, and potential explanatory variables with p < 0.1 in univariate analysis. Explanatory variables were initially identified through Chi-square, Student’s t-, and Mann–Whitney U tests and subsequently confirmed via univariable Cox proportional hazard models. Statistical significance was set at p < 0.05. All analyses were conducted using JMP version 13.2.0 (SAS Institute Inc., Cary, NC, USA) and SPSS version 26.0 (IBM, Armonk, New York, USA).

**References**

#E1. Ichinose M, Sugiura H, Nagase H, et al. Japanese guidelines for adult asthma 2017. Allergol Int 2017; 66: 163-189.

#E2. Chung KF, Wenzel SE, Brozek JL, et al. International ERS/ATS guidelines on definition, evaluation and treatment of severe asthma. Eur Respir J 2014; 43: 343-373.

#E3. Miller MR, Hankinson J, Brusasco V, et al. Standardisation of spirometry. Eur Respir J 2005; 26: 319-338.

#E4. American Thoracic Society/European Respiratory Society (2005). ATS/ERS recommendations for standardized procedures for the online and offline measurement of exhaled lower respiratory nitric oxide and nasal nitric oxide, 2005. Am J Respir Crit Care Med 2005; 171: 912-930.

# E5. Reddel HK, Taylor DR, Bateman ED, et al. An official American thoracic society/European respiratory society statement: asthma control and exacerbations: standardizing endpoints for clinical asthma trials and clinical practice. Am J Respir Crit Care Med 2009; 180: 59-99.
